# Over a Decade of *recA* and *tly* Gene Sequence Typing of the Skin Bacterium *Propionibacterium acnes*: What Have We Learnt?

**DOI:** 10.3390/microorganisms6010001

**Published:** 2017-12-21

**Authors:** Andrew McDowell

**Affiliations:** Northern Ireland Centre for Stratified Medicine, School of Biomedical Sciences, Ulster University, Londonderry BT47 6SB, UK; a.mcdowell@ulster.ac.uk

**Keywords:** *Propionibacterium acnes*, *recA*, *tly*, typing, phylogenetics, horizontal gene transfer, multiplex PCR, MLST, SLST, ribotyping, MLVA, whole genome sequencing

## Abstract

The Gram-positive, anaerobic bacterium *Propionibacterium acnes* forms part of the normal microbiota on human skin and mucosal surfaces. While normally associated with skin health, *P. acnes* is also an opportunistic pathogen linked with a range of human infections and clinical conditions. Over the last decade, our knowledge of the intraspecies phylogenetics and taxonomy of this bacterium has increased tremendously due to the introduction of DNA typing schemes based on single and multiple gene loci, as well as whole genomes. Furthermore, this work has led to the identification of specific lineages associated with skin health and human disease. In this review we will look back at the introduction of DNA sequence typing of *P. acnes* based on *recA* and *tly* loci, and then describe how these methods provided a basic understanding of the population genetic structure of the bacterium, and even helped characterize the grapevine-associated lineage of *P. acnes*, known as *P. acnes* type Zappe, which appears to have undergone a host switch from humans-to-plants. Particular limitations of *recA* and *tly* sequence typing will also be presented, as well as a detailed discussion of more recent, higher resolution, DNA-based methods to type *P. acnes* and investigate its evolutionary history in greater detail.

## 1. Introduction:

**Figure 1:**
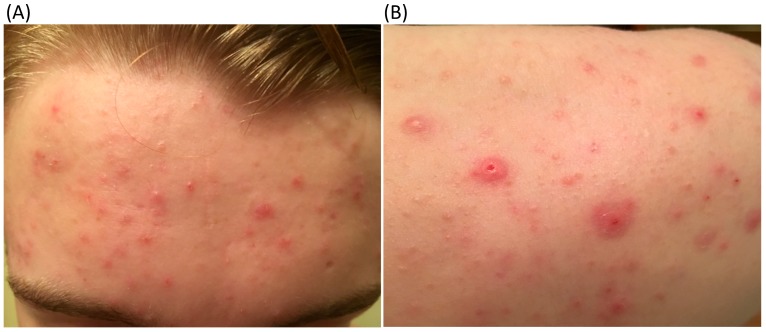
Moderate inflammatory and non-inflammatory acne lesions on the forehead (**A**) and shoulder (**B**) of a 14-year-old adolescent boy.

*Propionibacterium acnes* is a Gram-positive anaerobic bacterium and member of the ‘cutaneous’ group of human propionibacteria on the skin, although it can also be isolated from the oral cavity as well as the genitourinary and gastrointestinal tracts [1]. The organism is an opportunistic pathogen associated most notably with the common skin condition acne vulgaris after which it is named [2]. Acne is an inflammatory disease of the pilosebaceous follicle affecting the face, chest and back, primarily during adrenarche (Figure 1). 

Over the last 20 years, however, there has been an increasing recognition that we may have significantly underestimated the role of this bacterium in other human infections and clinical conditions. From the scientific literature, we now have a large body of evidence associating this bacterium with infections of indwelling medical devices, especially shoulder prostheses, as well as sarcoidosis, prostate cancer, back pain and fatal granuloma after soft tissue trauma [3,4,5,6,7]; further work is, however, required to prove whether the bacterium actually plays a role in the development of some of these conditions, especially lumbar disc herniation and prostate disease.

Within the last decade, we have also seen significant advances in our understanding of the intraspecies phylogeny of *P. acnes*. Distinct phylogroups have been discovered, and specific strains or sequence types (ST) associated with human health or disease revealed [8,9,10,11,12]. In this review we will initially focus on how DNA sequence typing schemes based on *recA* and *tly* loci were used to reveal the basic underlying phylogenetic structure of *P. acnes*. Specific issues or caveats with *recA* and *tly* sequence typing will then be highlighted, alongside a detailed description of newer, high resolution, molecular typing methods that have been developed to characterize the bacterium and better detail its phylogeny.

## 2. Introduction of *recA* and *tly* Gene Sequencing for Phylogenetic Analysis of *P. acnes*

Gene sequence analysis of *P. acnes* based on the non-ribosomal housekeeping (HK) gene *recA* (1047 bp; PPA1012) and the putative haemolysin/FtsJ-like methyltransferase gene *tly* (777 bp; PPA1396) was first described in 2005 as part of a larger study investigating the phylogenetic relationship between the two serotypes of *P. acnes*, known as types I and II [8]. Analysis of these genetic loci revealed that types I and II represented highly distinct phylogenetic groupings or divisions within the species. It also revealed further phylogenetic sub-divisions within the type I clade, designated types IB and IC, which displayed atypical reactions with monoclonal antibodies (mAb) originally developed for the identification of type I (QUBPa1) and type II (QUBPa2) strains [8,11,13]. Both antibodies, which target antigenically variable cell surface adhesins that bind dermatan sulphate (DsA1; DsA2) (QUBPa1) and a glycolipid-containing antigen (QUBPa2), show no significant reaction with the type IB lineage, but do react with type IC strains [8,11,14]; other type I strains, designated type IA, react with QUBPa1 but not QUBPa2. Recently, the DsA1 protein has also been shown to bind human fibrinogen [15]. Interestingly, just five months after the description of the *recA* and *tly* sequencing results, Cohen et al. [5] described three distinct pulsed field gel electrophoresis (PFGE) groups of *P. acnes* isolated from cancerous prostate tissue. We now know these pulsogroups actually correspond to the *recA* and *tly* types IA, IB and II [11,12]; in the same study, sequencing of the transcarboxylase 12S gene could differentiate pulsogroups I (type IA) and II (type IB) from group III (type II), but could not further subtype the type I clade. In 2008, application of *recA* gene sequencing was also instrumental in the identification of a new phylogenetic division within *P. acnes*, which was designated *recA* type III [9]. Strains of the type III clade, which were originally isolated from spine intervertebral disc material and now also human skin, have the capacity to form a filamentous-like cell morphology not observed with types I and II, and are also non-reactive with QUBPa1 and QUBPa2.

## 3. Choice of *recA* and *tly* Loci for Phylogenetic Analysis

The *recA* gene was chosen by McDowell et al. [8] since it is a valuable phylogenetic marker in bacterial systematics, and classifications based on *recA* have proved to be robust and consistent with those obtained by using rRNA genes [16]. Furthermore, protein-encoding HK genes can often provide greater phylogenetic resolution of very closely related organisms compared to the 16S rRNA gene due to a higher molecular clock speed, thereby generating a larger pool of informative sites for phylogenetic reconstructions [17]. Consistent with this, in the case of types I and II only one type-specific polymorphism at position 827 was identified within the 16S gene of the strains selected for analysis, versus 10 type-specific differences in *recA* [8]. The *recA* gene has also been successfully used for inter- and intra-species differentiation of organisms of the *Burkholderia cepacia* complex, *Agrobacterium* spp. and for typing of *Vibrio cholerae* isolates [18,19,20,21].

In the case of the *tly* gene, it was selected for analysis since it had a putative function as a cell surface/extracellular haemolysin/cytotoxin. In contrast to HK gene loci, such genes are often under strong positive selection for non-synonymous mutations and recombinational events by the host immune system leading to rapid diversification and increased discriminatory power for finer scale typing [22]; such ‘hyper-variable’ genes are often used to investigate very recent patterns of descent (short term epidemiology) and used as a complement to Multilocus Sequence Typing (MLST) analysis, as well as providing valuable information on the evolution of virulence loci. In the case of the *tly* gene, however, no clear evidence for diversifying selection exists upon analysis of multiple allele sequences, and this locus appears to have co-evolved with HK genes potentially indicating an important role in commensal existence for the bacterium [11,12]. The observation that the Tly protein family now needs to be redefined as an RNA-binding FtsJ-like methyltransferase involved in ribosomal biogenesis may be important in this context, indicating a dual function as a HK gene alongside a role as a haemolysin [12,23]. The *tly* gene is also present in other human propionibacteria, including *P. avidum*, *P. humerusii* and the recently described species *P. namnetense* [24].

## 4. Adoption of *recA* and *tly* Gene Sequencing as a Typing Tool for *P. acnes*

Prior to the description of *recA* and *tly* sequencing analysis of *P. acnes*, typing methods for the bacterium focused on fermentation, phage and serological reactions, DNA macrorestriction profiling by PFGE and Random Amplification of Polymorphic DNA analysis [25,26,27,28,29]. Soon after publication, researchers started to adopt *recA* and *tly*-based sequencing as a moderate resolution typing method, with the genes being utilized individually for single locus sequence typing (SLST), or concatenated for multilocus analysis. These genes have been used as typing tools to investigate whether the different phylogroups of *P. acnes* are associated with specific human infections or clinical conditions, including prosthetic joint and dental infections, sarcoidosis, prostate cancer and lumbar disc herniations highlighted earlier [30,31,32,33,34,35,36]. These sequence-based typing methods have also been used to genetically characterize *P. acnes* strains isolated from healthy human stomach mucosa [37]. Such studies started to provide initial evidence that differences may indeed exist in the association of specific phylogroups with disease, including strains of type IB and II with prosthetic joint infections and type I and II with prostate cancer. In addition to disease associations, characterization of *P. acnes* strains based on their *recA* and *tly* phylogenies has provided a framework onto which intraspecies genomic and proteomic characteristics can be mapped [38,39], as well as a broad range of putative virulence, biochemical and immunological properties [8,9,13,31,32,40]. A good example is the production of DsA1 and DsA2 adhesins by strains of type IA and IC, but not IB, II or III, and the differential production of CAMP-factors, in particular the abundant production of CAMP factor 1 by type IB and II strains, but not type IA [13,39]. Collectively, such studies started to reveal differences in pathogenic potential between phylogroups and their association with human disease.

## 5. *recA* and *tly* Gene Sequencing to Characterise *P. acnes* Type Zappe Strains

*recA* and *tly* gene sequence analysis has even been used to characterize strains of the grapevine-associated *P. acnes* lineage known as *P. acnes* type Zappe [41]. This new type of *P. acnes* appears to have adapted to an endophytic lifestyle, and is believed to have arisen from a human-to-plant interkingdom bacterial transfer approximately 7500 years ago during the domestication of grapevines [41]. Interestingly, although *P. acnes* type Zappe forms a distinct phylogenetic cluster from *P. acnes* upon 16S rDNA analysis, it groups with *P. acnes* type I strains based on *recA* and *tly* sequences. These discordant gene trees are likely to result from incomplete lineage sorting due to recent diversification. Furthermore, unlike *P. acnes*, the *recA* gene in *P. acnes* type Zappe appears to have lost its function during this unusual host switching event, possibly reflecting endosymbiosis, leading to the accumulation of a large number of non-synonymous mutations; in comparison, the *tly* and 16S rRNA genes of *P. acnes* type Zappe are much more conserved. These gene sequence characteristics, along with the detection of *P. acnes* type Zappe within plant tissues (bark, pith and fibres of Xylem vessels), provides evidence to support the endophyte nature of this bacterium and tight symbiosis with the plant.

## 6. Taxonomic Legacy

When the *recA* and *tly* typing methods were first described, our understanding of the underlying population genetic structure of *P. acnes* was poor. The identification of distinct genetic divisions and sub-divisions therefore provided a platform for more in depth study of the phylogenetic and taxonomic heterogeneity of *P. acnes*, and how this related to human health and disease. This has ultimately led to the recent proposal of the type I, II and III phylogroups as distinct subspecies known as *P. acnes* subsp. *acnes*, *P. acnes* subsp. *defendens* and *P. acnes* subsp. *elongatum*, respectively based on phylogenetic, genomic, phenotypic differences, as well as associations with different types of infections and clinical conditions including acne, progressive macular hypomelanosis (PMH), and prosthetic joint and soft tissue infections [42,43]. The cutaneous propionibacteria have now also been recently proposed as a novel genera, known as *Cutibacterium*, with *P. acnes* renamed as *Cutibacterium acnes* [44].

## 7. *recA* and *tly* Alleles Provide Hints of Conjugal Transfer of Large DNA Segments

The subsequent development of more recent molecular typing methods for *P. acnes* based on multilocus sequence typing (MLST) schemes (discussed later) combined with the growing number of available whole genome sequences, has provided newer, high resolution methods for detailed scrutiny of the population genetic structure of the bacterium. This has revealed much greater levels of phylogenetic structure within the type I phylogroup, enabling additional differentiation of strains into types IA_1_ and IA_2_, alongside the type IB and IC groups, and identification of clonal complexes (CC) and singleton strains [10,11,12,43,45,46]. These high-resolution MLST and whole genome sequencing (WGS) methods have, however, also revealed caveats with *recA* and *tly* sequencing, since the combination of *recA* and *tly* allele sequences originally used to identify the type IB phylogroup have now also been found in a subset of clearly defined type IA_1_ strains from CC4 (MLST_8_ nomenclature), all strains from the IA_2_ lineage, and the aberrant type IA_1_ singleton strain SK187 which displays a mosaic of genes found in other type I lineages, including type IB [10,14,38,45]. 

**Figure 2:**
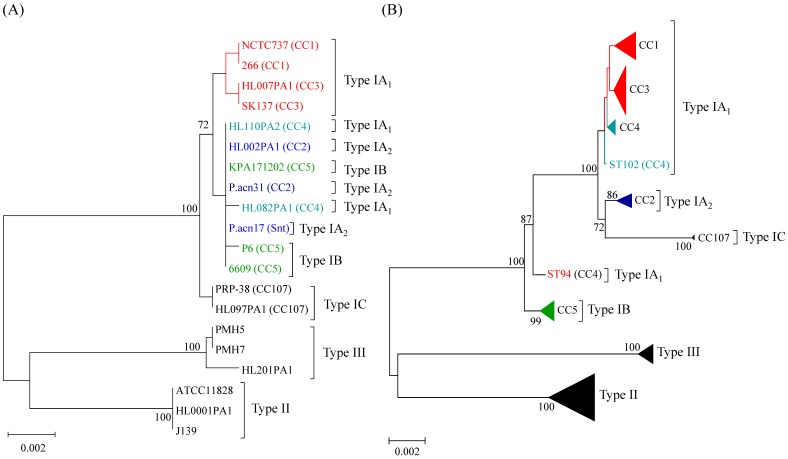
Minimum evolution phylogenetic trees of concatenated *recA* and *tly* sequences (1824 bp) from isolates selected to represent all known phylogroups (**A**), and concatenated gene sequences (4235 bp) from all current STs in the MLST_8_ database (**B**); the latter is updated from [43] with STs. Bootstrapping statistics were performed using 500 data sets, and only bootstrap values ≥70% are shown. Clonal complexes (CC) are indicated. An overlapping cluster of strains from CC4 (type IA_1_), CC2 (type IA_2_) and CC5 (type IB) can clearly be seen based on analysis of *recA* and *tly* sequences (**A**), but on MLST_8_ analysis strains from these different phylogroups form separate, distinct clusters with high bootstrap values (**B**).

This is illustrated in Figure 2A, which demonstrates phylogenetic clustering of strains crossing these different genetic distances based on concatenated *recA* and *tly* gene sequences (1824 bp) (the same clustering of strains is seen with single *recA* and *tly* trees; Figure S1), yet clear, non-overlapping clustering of these phylogroups when high resolution MLST_8_ analysis is applied (Figure 2B). These results help to explain why a sub-set of strains presumptively identified as type IB based on *recA* and *tly* analysis displayed reaction with the type IA mAb QUBPa1 [11,14].

**Figure 3:**
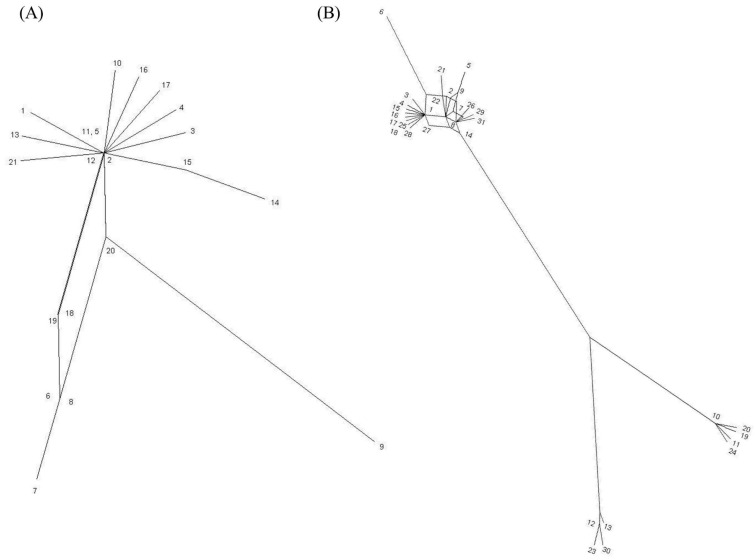
Split decomposition analysis of all current *recA* (**A**) and *tly* (**B**) allele sequences. *recA* allele sequences (753 bp) were taken from the MLST_9_ database [53] and *tly* allele sequences (777 bp) from the MLST_8_ database [54]; the latter is updated from [12] with new allele sequences. No evidence of statistically significant recombination was identified in either genes using the Phi test (*p* = 0.135 for *recA*; *p* = 0.735 for *tly*), although some interconnected pathways were present.

*P. acnes* has a clonal population structure and is in linkage disequilibrium [11,12]. The organism has a high degree of sequence conservation, although recombination events are still evident in the history of the bacterium. Due to this, *P. acnes* is a candidate organism for the development of phylogenetically accurate SLST schemes. Interestingly, phylogenetic network analyses reveals only the *tly* sequences conforming to a very clear tree-like structure, although the Phi test for recombination is not statistically significant for either loci (Figure 3). Furthermore, phylogenetic trees based on the analysis of the individual genes are congruent (Figure S1). The discovery of linked alleles across different phylogenetic lineages does, however, suggest conjugal transfer and homologous recombination of very large DNA segments; these genes comprise a genomic fragment that stretches to at least 420 Kbp (KPA171202 coordinates 1095280-1515117). This scenario would help to explain why *recA* and *tly* sequences generate congruent phylogenies.

Further support also comes from the identification of *camp5* (KPA171202 coordinates 1305348-1306193) and *gms* (KPA171202 coordinates 1503535-1504578) alleles that are also shared between CC4 and types IA_2_ and IB, and located within the same segment [10]. Collectively, this would tentatively suggest that multiple conjugation events of unusually large chromosomal replacements have shaped the genome dynamics of the type I clade; such large chromosomal replacements have also been observed with *Staphylococcus aureus* and *Streptococcus agalactiae* [47,48]. While such events are assumed to be rare due to the large size of the fragments, they may have had a dramatic effect on the evolution of the bacterium. More detailed sequence-based analyses will, however, be required to confirm this possibility. Alongside this, *P. acnes* has a flexible gene pool with the presence and absence of different island-like genomic regions with aberrant G+C content and flanking insertion sequences that encode putative virulence factors and traits potentially associated with fitness and niche adaptation [38,49,50]. 

Rates of recombination also appear to differ throughout the population, and the association of alleles appears less significant when distinct phylogroup populations are considered [11,12]. Reduced rates of recombination between the different subsp. of *P. acnes* may indicate ecological differences within the body since members of the same habitat are more likely to undergo recombination events (sympatric speciation) [51]. Against this background, it is interesting that only type II strains have an active CRISPR/Cas system, making type I and III strains potentially more susceptible to horizontal gene transfer (HGT) of fitness and virulence traits [46,52].

## 8. Newer Molecular Typing Methodologies for *Propionibacterium acnes*

As the *recA* and *tly* loci cannot reliably identify type IB strains from those related to the type IA_1_ sub-clade corresponding to CC4, or strains of type IA_2_, their application as a typing tool has limitations; the methods are still valid, however, for the identification of almost all other type IA_1_ strains, as well as those from the type IC, II and III phylogroups. Since the *recA* and *tly* sequencing approaches were originally reported over 10 years ago, a wide range of newer DNA-based typing methods have been described with distinct advantages and disadvantages to one another; most of these are still superior to the original *recA* and *tly* typing methods in terms of its moderate resolving power. These key methods include:

### 8.1. MLST Schemes

MLST is based on indexing genetic variation within multiple HK genes, normally seven [55]. Unique alleles for each loci are given an arbitrary number, and the different combinations of these alleles, known as the allelic profile, assigned a ST number. The allelic profile therefore does not take into consideration the number of nucleotide differences between alleles, and this lack of weighting in regard to sequence divergence helps to correct for HGT events which can distort phylogenetic signals [55]. In addition to concatenated DNA sequence analysis which provides minimal information of ancestry or patterns of descent, MLST enables isolates to also be grouped or clustered based on sharing a defined number of alleles (usually ≥6/7) with at least one other member of the group, thus generating non-overlapping CCs and predicting founding genotypes. This type of analysis, which is performed using an algorithm such as eBURST, provides the opportunity to infer appropriate patterns of evolutionary descent between isolates over short timescales [56]. 

**Figure 4:**
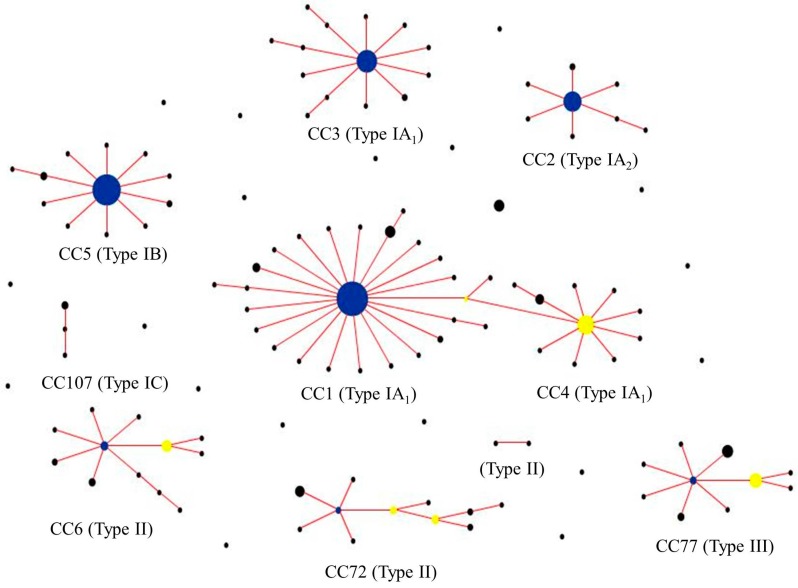
eBURST population snapshot of the current *P. acnes* MLST_8_ database. To date, a total of nine clonal complexes, where the isolates share 7/8 loci with at least one other ST in the group, and 19 singletons have been identified from the analysis of over 400 isolates. Founding genotypes are highlighted in blue and sub-founders in yellow. The frequency of each ST is indicated by circle size.

Two independent MLST schemes and associated, publically available, databases for *P. acnes* have now been developed based on completely different sets of protein-encoding gene loci [10,11]. These schemes provide high resolution typing of the bacterium and generate phylogenies which are essentially congruent with those based on WGS analysis. One scheme highlighted earlier in the review, and based on the analysis of eight loci (MLST_8_; 4253 bp), was originally developed at the University of Warwick and then modified by researchers at Queen’s University, Belfast to include the *camp2* and *tly* genes [11] (Figure 2B); the database for this scheme can be found at *Propionibacterium acnes* MLST Databases [54]. A second scheme, which was based on nine loci and corresponding amplification and sequencing primers developed at the University of Bath, was fully adopted by researchers at Orebro University (4536 bp) [57]. Researchers at Aarhus University also utilized this method but replaced the *cob* gene with *recA* (known as MLST_9_; 4233 bp) and also developed a database for the scheme which can be found at Multi Locus Sequence Typing [53]. Both MLST_8_ and MLST_9_ schemes are generally concordant in how they cluster strains into different CCs, but some differences do exist in the resolution of particular strains within these CCs. These CC equivalencies and specific strain differences have been detailed elsewhere [11]. At the time of writing, the MLST_8_ database comprises 127 distinct STs with nine CCs superimposed on a background of 19 singletons in sequence space (Figure 4). The MLST_9_ database comprises 125 STs.

**Figure 5:**
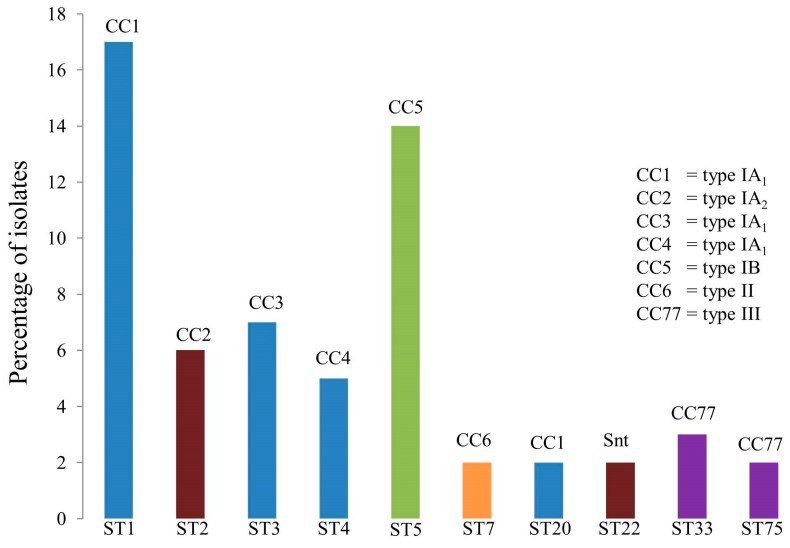
Current top 10 most populous STs within the *P. acnes* MLST_8_ isolate database. Clonal complexes (CC) are also indicated.

Being a DNA-sequencing based method, MLST offers portability over gel-based typing approaches, and is an extremely valuable and stable system for global epidemiology and genetic population studies. Indeed, using these methods we and others have shown that *P. acnes* has an epidemic population structure, with highly successful clonal lineages that are globally disseminated [e.g., ST1 and ST3 (type IA_1_), ST2 (type IA_2_) and ST5 (type IB) based on MLST_8_ analysis] within the human population [11,12] (Figure 5); some of these epidemic lineages appear to be permanent members of the skin microbiota and are associated with acne [11,12]. 

Despite all its advantages, the key downsides of MLST are its labor and time consuming nature as well as expense, especially when analyzing large numbers of isolates. In an attempt to circumvent these problems, and to streamline the MLST workflow, it has been shown that only four of the eight MLST_8_ gene loci (*aroE*, *guaA*, *camp2*, *tly*) need to be sequenced to correctly predict phylogroup, CC and in most cases ST from the full MLST profiles available in the database [12]. This is only possible because of the clonal nature of *P. acnes*, and the relatively limited number of genotypes that are found. 

### 8.2. Multiplex PCR

A set of three separate PCR assays for phylogroup typing of *P. acnes* was originally described in 2006 by Shannon et al. [58]; this method was only capable of resolving type IA, IB and II strains, providing no data on type IA_1_, IA_2_, IC or type III status. More recently, a multiplex touchdown PCR typing method for *P. acnes* isolates that provides unambiguous identification of all the main phylogroups (type IA_1_, IA_2_, IB, IC, II and III) in a single reaction has been described [59]. The method utilizes six primer sets that target the 16S rRNA gene to confirm species identify (all isolates), ATPase (types IA_1_, IA_2_, and IC), *sodA* (types IA_2_ and IB), *atpD* (type II), and *recA* (type III) HK genes, as well as a Fic family toxin gene (type IC); the HK loci on which the multiplex assay was developed are from the MLST_8_ scheme where multiple alleles of each gene are available for analysis and identification of type-specific single nucleotide polymorphisms (SNPs) for primer design. Phylogroup identification is based on the visual pattern of reaction with the different primer sets. The key advantage of this 6-plex PCR assay is that it provides a rapid, high-throughput, and technically undemanding typing method for epidemiological and phylogenetic investigations. The multiplex assay also provides a simple way to identify potentially novel taxa due to atypical or non-typeable PCR patterns that could arise, for example, when new alleles or allele combinations are encountered in new STs. A limitation of the multiplex method is that it only resolves isolates to the phylogroup level; if very high resolution typing is required then other methods, such as MLST or WGS, should be carried out. These latter methods, however, only provide significantly more information for type IA_1_ and type II phylogroups due to their greater genetic heterogeneity and deeper level of phylogenetic structure relative to types IA_2_, IB, IC, and III. In the latter case, the phylogroups represent relatively tight phylogenetic clusters (only one CC for each phylogroup) with a limited number of STs, some of which are highly dominant in the population and widely disseminated (Figure 4 and Figure 5). 

The multiplex assay has now been described for the typing of *P. acnes* isolates from patients with acne [60,61,62], PMH [63], wound exudates and abscesses [62], microdiscectomy tissue [64], central nervous system and prosthetic joint infections [65], and other clinical conditions [60]. It has also been used for high throughput typing of *P. acnes* isolates from the pre-operative skin and surgical wound of patients undergoing spinal operations; this study was a large randomized controlled trial investigating the effects of povidone-iodine-alcohol (PVI) and chlorhexidine gluconate-alcohol on surgical wound contamination compared to PVI alone [66]. The validation of the multiplex method by a number of independent groups has led to the conclusion that it is a robust and valid method for *P. acnes* typing [62]. 

### 8.3. Ribotyping

A ribotyping scheme for *P. acnes*, based on analysis of the 16S rRNA gene (~1450 bp; positions 29-to-1483 bp), has been described by Fitz Gibbon et al. [46]; novel ribotypes (RT) in this SLST scheme were assigned based on at least one unique SNP. Upon Sanger sequencing of amplicons obtained from genomic DNA pooled from pilosebaceous units (‘pores’) on the nose of acne and control subjects, more than 11K RTs were identified, although only a very small number appear abundant in the skin with the minor RTs representing singletons. Compared to RT1, the most abundant of the RTs, all other defined RTs have ≥99% sequence identify which is in keeping with the highly conserved nature of the 16S rRNA locus at the intraspecies level. Despite this, the limited number of SNPs available can differentiate the main type I, II and III phylogroups from one another, as well as type IA_1_ from IA_2_ strains which have unique RTs in keeping with their distinct phylogenies (Table 1). 

Within the type IA_1_ clade, strains corresponding to CC4 by MLST_8_ analysis (equivalent to CC31 by MLST_9_) can also be resolved, but a limitation of the method is that strains from CC1 and CC3 by MLST_8_ analysis (equivalent to CC18 and CC3, respectively by MLST_9_), as well as type IB and IC strains, cannot be separated as they share RT1 and RT5 (Table 1). As the ribotyping approach has specific strain resolution limitations, this is the main disadvantage to its use. As it is only based on one locus, the data is also not amenable to allelic profile clustering. It does, however, significantly reduce Sanger sequencing costs compared to MLST analysis of isolates while retaining the advantage of DNA sequence portability and stability between laboratories. Currently, there is no public database for the *P. acnes* ribotyping scheme. 

### 8.4. High Resolution SLST (HR-SLST)

**Figure 6:**
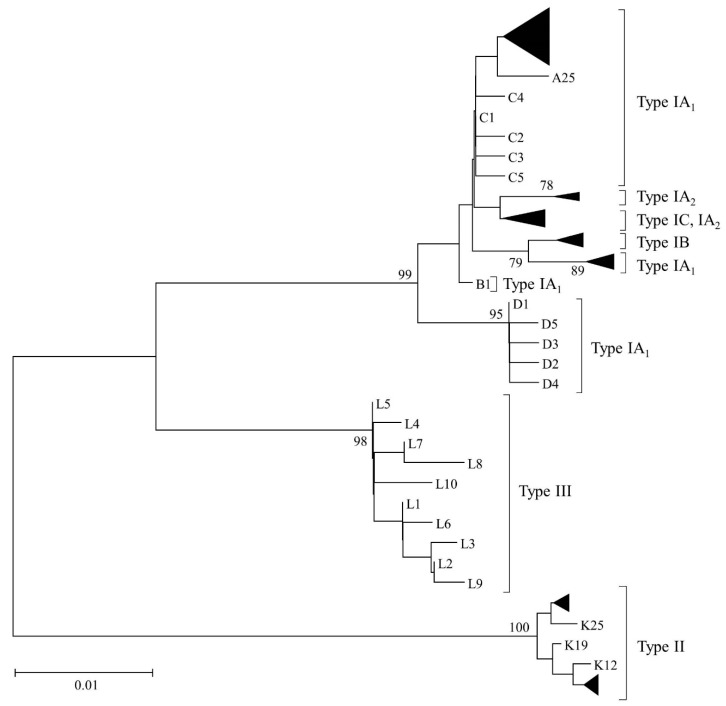
Minimum evolution tree of the current 112 STs from the 483–497 bp HR-SLST target locus. Sequences are from the SLST database at SLST for *Cutibacterium acnes* (formerly *Propionibacterium acnes*) [68]. Bootstrapping statistics were performed using 500 data sets, and only bootstrap values ≥70% are shown.

**Figure 7:**
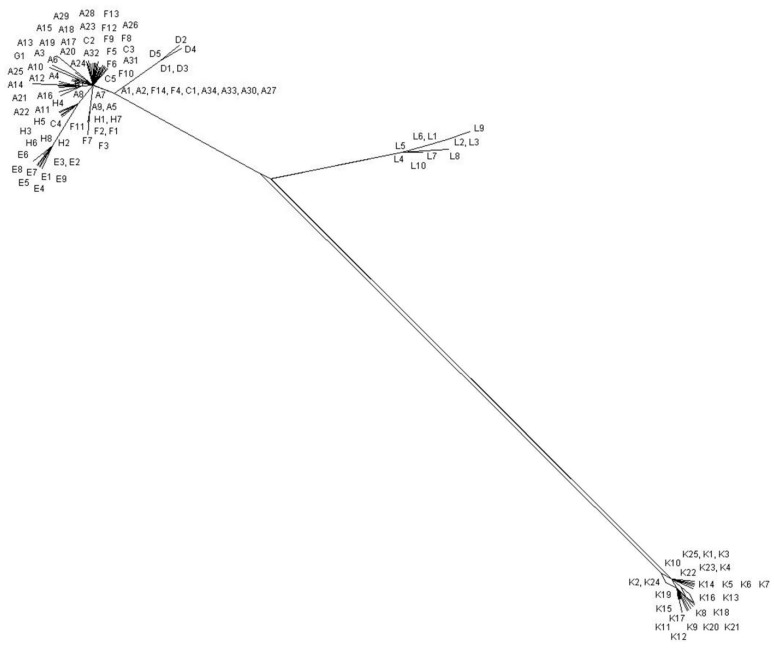
Split decomposition analysis of all current SLST allele sequences (483–497 bp) from the HR-SLST database [68]. No evidence of statistically significant recombination was identified using the Phi test (*p* = 0.399). Updated from [59] with new allele STs.

The development of a high resolution SLST (HR-SLST) scheme for *P. acnes* is clearly desirable since it would reduce the expense and workflow involved in MLST analysis, alongside its ability to directly analyze complex biological samples for *P. acnes* population structure. While *P. acnes* is an appropriate choice for the development of such a method, the low level of genetic variability found amongst the HK genes of this clonal organism does make such a task difficult. To address this issue, Scholz et al. [67] utilized novel python scripts to ‘mine’ the available whole genome sequences of all known *P. acnes* phylogroups, and identify sufficiently variable target loci on which a novel SLST scheme could be developed. This computational analysis pinpointed a 483–497 bp sequence which differentiated strains to a level of resolution approaching that obtained by MLST (Figure 6, Table S1); although the pattern of phylogroup clustering within the type I clade varies somewhat from that obtained by MLST and WGS analysis. This sequence is located immediately upstream of the CAMP factor 1 gene (PPA1340), and partially overlaps with a number of hypothetical genes [67]. The success of this locus suggests that recombination events are extremely limited in its history, and this is confirmed by phylogenetic network analysis which shows a tree-like structure, and a non-significant Phi test for recombination (Figure 7). The SLST method uses a letter and number-based nomenclature for ST assignment and a database for the scheme can be found at SLST for *Cutibacterium acnes* (formerly *Propionibacterium acnes*) [68]. 

In addition to isolate analysis, the SLST method has also been used to examine the *P. acnes* community structure within skin swab samples taken from various body sites of a healthy individual [67] and patients with PMH [69] via Roche 454 pyrosequencing, which provides reads sufficiently long to cover the target sequence; this metagenomic approach enables mixed strain populations of the bacterium to be analyzed. The method can, however, be adapted to run on other next generation sequencing (NGS) platforms, such as Illumina using pair-end reads (2 × 300 bp). Similar to ribotyping, the SLST approach will reduce Sanger sequencing costs compared to MLST, and is a portable and stable system, although as it is only based on one locus the data is also not amenable to allelic profile clustering.

### 8.5. Multiple Locus Variable Number of Tandem Repeat (VNTR) Analysis (MLVA)

MLVA is a typing method for bacteria that is based on the variable number of tandem repeats (VNTR) present in multiple loci. These hypervariable loci are short sequences of repetitive DNA that arise due to slipped strand mispairing during DNA replication, and frequently vary between strains from the same species. An MLVA scheme for *P. acnes* based on the analysis of 13 VNTRs (MLVA_13_) has been described by Hauck et al. [70]. These included 10 large VNTRs with repeat units ranging from 14–31 bp, and three small VNTRs with 6 bp size repeats. Like MLST, MLVA generates an allelic profile which in this case corresponds to the number of repeats at each locus, and a specific MLVA type number. The selected VNTR markers in *P. acnes* are present in genes that include putative HtaA domain and adhesin proteins, fibrinogen- and penicillin-binding proteins and hypotheticals, as well as an intergenic region [70]. With the MLVA_13_ scheme, clustering of *P. acnes* isolates was found to be in good agreement with phylogenies based on WGS analysis, although a small number of discrepancies were observed. An advantage of this method is that it is relatively cheap, fast and easy to perform but portability can be a significant issue when amplicons are sized as banding patterns by conventional electrophoresis [71]; this can be greatly improved by capillary electrophoresis on an automated DNA sequencing instrument. Another potential issue with this method is the stability of the VNTR markers, although the small VNTRs used in this scheme appear to be stable upon repeat testing [70]. To date, the *P. acnes* MLVA scheme is yet to be utilized by the wider research community. The *P. acnes* MLVA database is scheduled to be released shortly at MLVAbank for Microbes Genotyping [72].

### 8.6. Whole Genome Sequencing

High resolution MLST of *P. acnes* generates phylogenies that, in general, match those obtained by WGS analysis. When analyzing a large number of isolates, however, it is an expensive typing methodology, especially with Sanger sequencing costs which are generally in the range of £3-to-£5 per read depending on the service provider. As the costs of NGS have continued to fall, and are comparable per isolate to MLST, rapid and high quality WGS analysis of bacterial strains is now increasing common for epidemiological investigations [73,74]. 

Unlike traditional MLST which is normally based on seven loci, WGS provides the opportunity to develop scaled up core-genome MLST (cgMLST) schemes, or a whole-genome MLST (wgMLST) approach, where all the equivalent loci between isolates are compared (core + accessory); this is based on de novo assembly of sequences to a reference genome [75]. As an alternate to the gene-by-gene approach, the phylogenetic relationships between isolates can also be based on the analysis of differences in genome core SNPs, which offers fine resolution but requires a reference sequence; this method is based on mapping short reads to a reference genome. To date, over 120 *P. acnes* genomes have been completed or are at the draft stage [76], and both core SNP and cgMLST analysis (based on the concatenated sequence of 76 HK genes) have been applied to this dataset to create whole genome trees [38,43,45,46]. 

WGS is the ultimate typing tool providing information on the full gene complement and genomic rearrangements. The gene-by-gene approach does, however, enable a hierarchical approach to typing where the number of genes analyzed is selected on the level of resolution required. Ultimately, WGS will likely replace other current molecular methods of *P. acnes* typing as costs continue to decline and bench top NGS sequencers become commonplace; the genome is also relatively small for sequencing (2.5 Mbp). Furthermore, various online web servers now provide software that will generate MLST profiles from whole genome data, such as Bacterial Isolate Genome Sequence Database (BIGSdb) [77,78] and the Centre for Genomic Epidemiology [79]. A key issue to address moving forward, however, is agreement on the optimal number of genes to use for WGS typing of *P. acnes* while maintaining epidemiological concordance; this would help to standardize interlaboratory comparisons. 

## 9. Typing Algorithm

**Figure 8:**
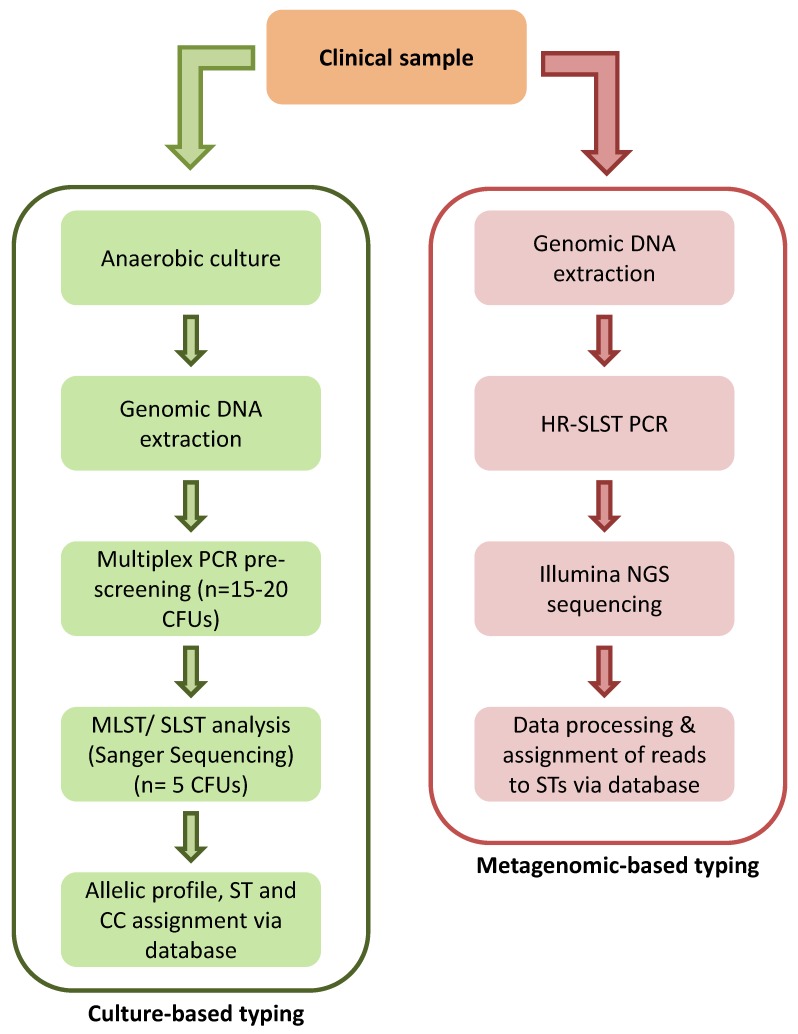
Algorithm for culture and non-culture-based typing pathways for *P. acnes*.

The level of resolution required for a typing scheme depends on the epidemiological questions being addressed. For *P. acnes* we currently use the algorithm highlighted in Figure 8 as our normal approach to typing of the bacterium. For culture-based investigations, multiple colonies (*n* = 15–20) from a sample are rapidly pre-screened by multiplex PCR as a first line typing method, before a further subset of these isolates (*n* = 5) are analyzed by high resolution MLST/ SLST if required. This approach not only helps to estimate the degree of phylogroup heterogeneity within a sample, but also maximizes the number of genetically diverse isolates selected for additional high resolution typing while keeping costs down. For non-culture-based typing, we utilize HR-SLST for direct metagenomic analysis based on the Illumina MiSeq platform. This approach enables the relative abundance of different pylogroups and SLST genotypes within a sample to be determined, but is more technically demanding.

## 10. Conclusions

In conclusion, the introduction of *recA* and *tly* gene sequence typing of *P. acnes* over 10 years ago proved an important first step in dissecting the underlying phylogenetic structure of this bacterium and the association of different genetic groupings with human health and disease. Since then, many newer molecular-based typing methodologies for *P. acnes* have been introduced. In particular, multiplex PCR and MLST/ HR-SLST, either individually or combined, now represent popular choices for typing, providing valuable and accurate information of the genetic population structure of the bacterium within clinical samples.

## Figures and Tables

**Table 1:** Correlation between ribotypes, phylogroups and MLST CCs.

		MLST_8_	MLST_9_
Ribotype	Phylogroup	Clonal Complex	Clonal Complex
1, 5, 532	IA_1_	CC1	CC18
1, 4, 5	IA_1_	CC3	CC3
8	IA_1_	CC4	CC31
3, 16	IA_2_	CC2	CC28
1	IB	CC5	CC36
5	IC	CC107	Singletons
2, 6	II	CC6/CC72	CC60
9	III	CC77	ND

## Supplementary Materials

The following are available online at http://www.mdpi.com/2076-2607/6/1/1/s1.

Supplementary File 1
