# Supplementary material for: Over a Decade of recA and tly Gene Sequence Typing of the Skin Bacterium Propionibacterium acnes: What Have We Learnt?
Source: Microorganisms. 2017 Dec 21;6(1):1. doi: 10.3390/microorganisms6010001 (PMC5874615; doi:10.3390/microorganisms6010001)
Supplement: Supplementary File 1 [file microorganisms-06-00001-s001.pdf]

Figure S1. Minimum evolution phylogenetic trees for *recA* (A) and *tly* (B) sequences from isolates selected to represent all known phylogroups. Bootstrapping statistics were performed using 500 data sets, and only bootstrap values ≥ 70% are shown. The *recA* and *tly* sequences from the sister species *Propionibacterium humerusii* were used as an outgroup to root the trees (CC = Clonal Complex; Snt = singleton).

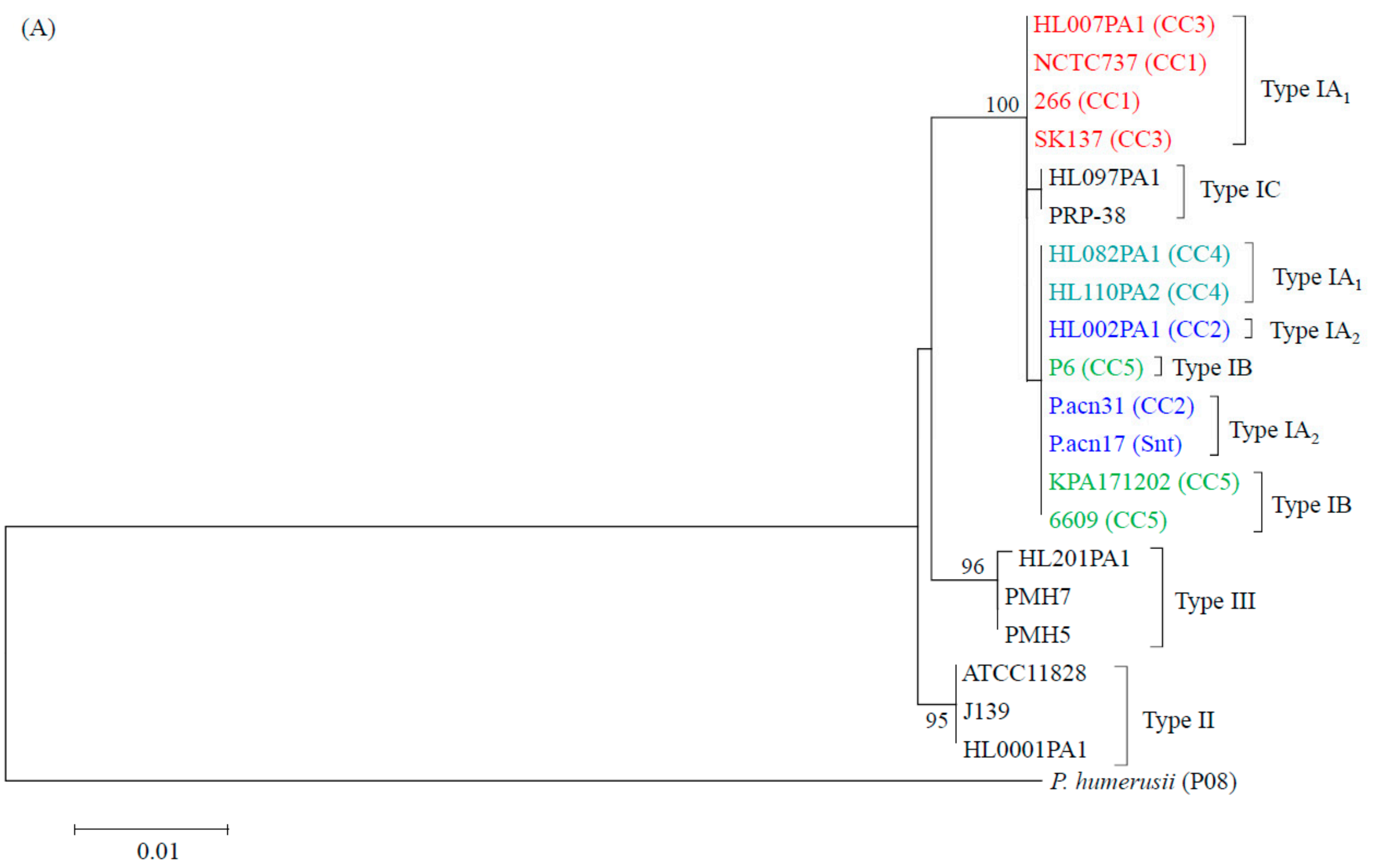

(B)

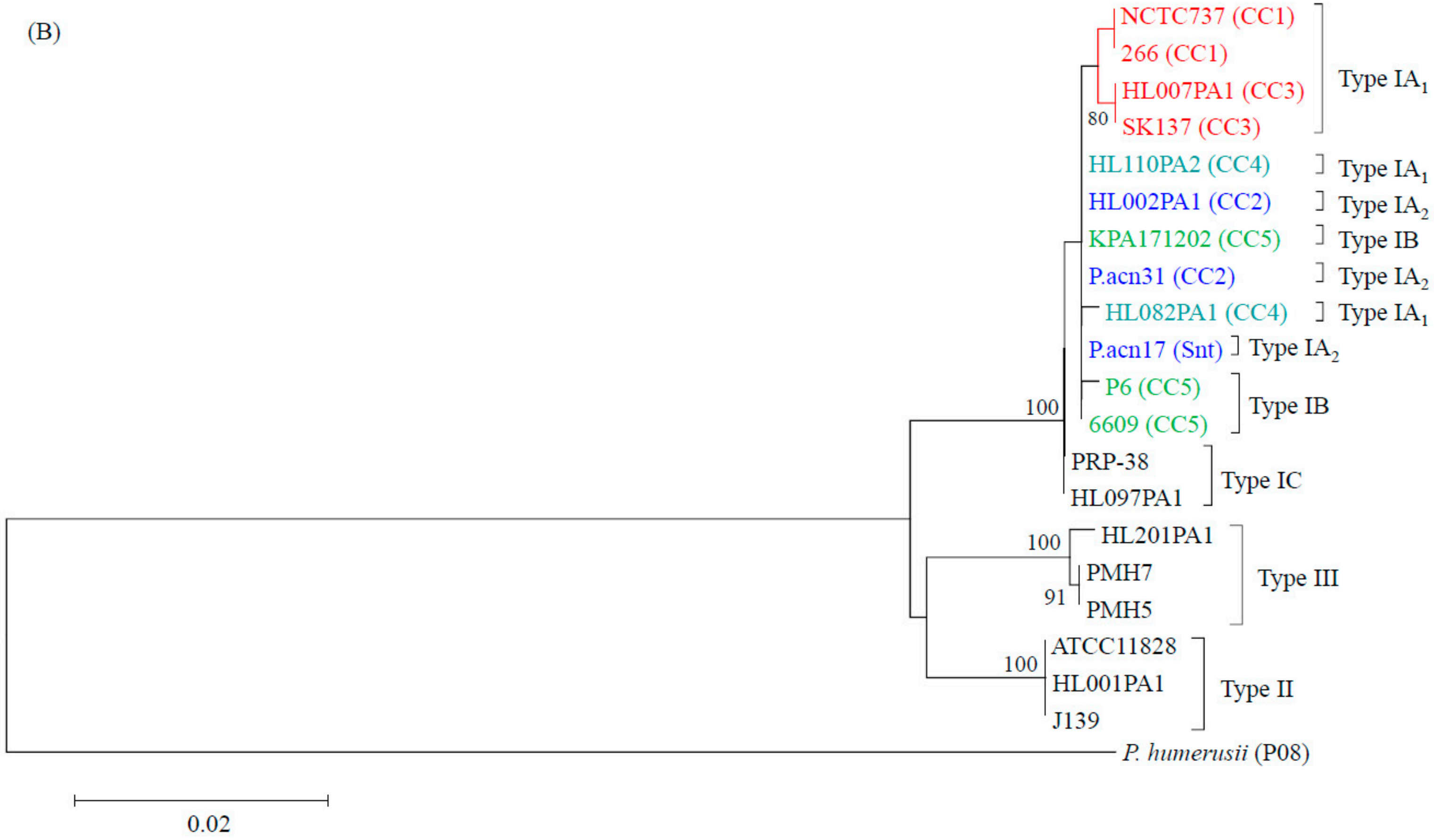
NCTC737 (CC1)
266 (CC1)
HL007PA1 (CC3)
SK137 (CC3)
Type IA1
80
HL110PA2 (CC4)
Type IA1
HL002PA1 (CC2)
Type IA2
KPA171202 (CC5)
Type IB
P.acn31 (CC2)
Type IA2
HL082PA1 (CC4)
Type IA1
P.acn17 (Snt)
Type IA2
P6 (CC5)
6609 (CC5)
Type IB
100
PRP-38
HL097PA1
Type IC
100
HL201PA1
PMH7
91
PMH5
Type III
100
ATCC11828
HL001PA1
J139
Type II
P. humerusii (P08)
0.02

Table S1. Updated MLST and SLST data for *P. acnes* isolates from complete and draft WGS projects and previously published studies.

				MLST$_8$ scheme[g]			
**Isolate** [a]	**Region**	**Source**	**Phylogroup**	**Allelic profile**	**ST**	**CC**	**SLST type**[h]
NCTC737[b]	UK	Acne	IA_1	1-1-1-3-1-1-1-1	1	1	A1
HL072PA2	USA	Acne	IA_1	1-1-1-3-1-1-1-1	1	1	A6
HL072PA1	USA	Acne	IA_1	1-1-1-3-1-1-1-1	1	1	A6
J165	USA	Skin	IA_1	1-1-1-3-1-1-1-1	1	1	A1
HL013PA2	USA	Acne	IA_1	1-1-1-3-1-1-1-1	1	1	A1
HL087PA2	USA	Acne	IA_1	1-1-1-3-1-1-1-1	1	1	A1
HL063PA1	USA	Skin	IA_1	1-1-1-3-1-1-1-1	1	1	A1
HL027PA2	USA	Skin	IA_1	1-1-1-3-1-1-1-1	1	1	A1
HL002PA3	USA	Acne	IA_1	1-1-1-3-1-1-1-1	1	1	A2
HL002PA2	USA	Acne	IA_1	1-1-1-3-1-1-1-1	1	1	A2
HL046PA2	USA	Acne	IA_1	1-1-1-3-1-1-1-1	1	1	A2
HL036PA3	USA	Skin	IA_1	1-1-1-3-1-1-1-1	1	1	A2
HL036PA2	USA	Skin	IA_1	1-1-1-3-1-1-1-1	1	1	A2
HL036PA1	USA	Skin	IA_1	1-1-1-3-1-1-1-1	1	1	A2
5U42AFAA[c]	Canada	ND	IA_1	1-1-1-3-1-1-1-1	1	1	A1
KPL2003[c]	USA	ND	IA_1	1-1-1-3-1-1-1-1	1	1	A1
DSM1897	USA	ND	IA_1	1-1-1-3-1-1-1-1	1	1	A1
NTS_31306190	France	Bone tissue	IA_1	1-1-1-3-1-1-1-1	1	1	A1
12.1.R1	Denmark	[10]	IA_1	5-1-1-3-1-1-1-1	20	1	A1
15.2.L1	Denmark	[10]	IA_1	1-1-1-3-1-1-1-1	1	1	A1
26.1.L1	Denmark	Acne	IA_1	1-1-1-3-1-1-1-1	1	1	A1
27.1.R1	Denmark	[10]	IA_1	1-1-1-3-1-1-1-1	1	1	A1
32.1.L2	Denmark	Skin	IA_1	1-1-1-3-1-1-1-1	1	1	A1
37.1.L1	Denmark	Acne	IA_1	1-1-1-3-1-1-1-1	1	1	A1
43.1.L1	Denmark	Acne	IA_1	1-1-1-3-1-1-1-1	1	1	A1
44.1.L1	Denmark	Acne	IA_1	1-1-1-3-1-1-1-1	1	1	A1
46.1.L1	Denmark	Acne	IA_1	1-1-1-3-1-1-1-1	1	1	A1

48.1.L1	Denmark	Skin	IA_1	1-1-1-3-1-1-1-1	1	1	A1
49.1.L1	Denmark	Skin	IA_1	1-1-1-3-1-1-1-1	1	1	A1
50.1.L1	Denmark	Skin	IA_1	1-1-1-3-12-1-1-1	126	1	A1
52.1.L4	Denmark	Skin	IA_1	1-1-1-3-1-1-1-1	1	1	A1
HL096PA3	USA	Skin	IA_1	1-1-1-3-1-1-3-1	8	1	A1
HL020PA1	USA	Acne	IA_1	1-1-1-3-1-1-4-1	9	1	A1
HL005PA2	USA	Skin	IA_1	1-1-10-3-1-1-6-1	15	1	A1
HL005PA3	USA	Skin	IA_1	1-1-10-3-1-1-1-1	16	1	A1
266	Germany	Pulmonary	IA_1	5-1-1-3-1-1-1-1	20	1	A1
HB	France	Skin	IA_1	5-1-1-3-1-1-1-1	20	1	A1
12.1.L1	Denmark	[10]	IA_1	5-1-1-3-1-19-1-1	127	1	A1
51.1.L1	Denmark	Skin	IA_1	1-1-1-3-1-1-1-1	1	1	A3
HL099PA1	USA	Acne	IA_1	1-1-1-3-1-1-2-2	3	3	C1
HL056PA1	USA	Skin	IA_1	1-1-1-3-1-1-2-2	3	3	C2
HL096PA2	USA	Skin	IA_1	1-1-1-3-1-1-2-2	3	3	C1
HL053PA1	USA	Acne	IA_1	1-1-1-3-1-1-2-2	3	3	C2
HL074PA1	USA	Skin	IA_1	1-1-1-3-1-1-2-2	3	3	C2
HL007PA1	USA	Skin	IA_1	1-1-1-3-1-1-2-2	3	3	C1
HL043PA1	USA	Acne	IA_1	1-1-1-3-1-1-2-2	3	3	C1
HL043PA2	USA	Acne	IA_1	1-1-1-3-1-1-2-2	3	3	C1
HL083PA1	USA	Acne	IA_1	1-1-1-3-1-1-2-2	3	3	C1
HL096PA1	USA	Acne	IA_1	1-1-1-3-1-1-2-2	3	3	C1
15.1.R1	Denmark	[10]	IA_1	1-1-1-3-1-1-2-2	3	3	C1
KPL2009[c]	USA	ND	IA_1	1-1-1-3-1-1-2-2	3	3	C1
HL005PA1	USA	Skin	IA_1	1-1-1-3-1-1-5-2	11	3	C2
HL038PA1	USA	Acne	IA_1	1-1-1-3-1-1-2-4	10	3	C1
HL078PA1	USA	Skin	IA_1	1-1-1-3-1-14-2-3	14	3	B1
HL045PA1	USA	Acne	IA_1	1-10-1-3-1-1-2-2	17	3	C2
SK137	USA	Skin	IA_1	8-1-1-3-1-1-2-5	18	3	C1
FZ1/2/0	Hungary	Skin	IA_1	1-1-1-3-1-1-2-3	31	3	B1
C1	Japan	Sarcoidosis	IA_1	1-1-1-3-1-1-22-2	115	3	A5

NTS_2004_10708	France	Bone tissue	IA_1	1-1-1-3-1-1-1-1	1	1	A26
KPL1854[c]	USA	ND	IA_1	1-1-1-3-1-17-2-2	120	3	C3
SK182	USA	Skin	IA_1	1-13-1-3-1-1-2-5	29	S	C1
HL025PA1	USA	Skin	IA_1	1-1-1-3-1-1-8-6	4	4	D1
HL086PA1	USA	Skin	IA_1	1-1-1-3-1-1-8-6	4	4	E4
HL092PA1	USA	Acne	IA_1	1-1-1-3-1-1-8-6	4	4	E1
HL110PA1	USA	Acne	IA_1	1-1-1-3-1-1-8-6	4	4	E1
HL053PA2	USA	Acne	IA_1	1-1-1-3-1-1-8-6	4	4	E1
30.2.L1	Denmark	[10]	IA_1	1-1-1-3-1-1-8-6	4	4	D1
MB3007[c]	USA	ND	IA_1	1-1-1-3-1-1-8-6	4	4	D1
NTS_2003_1719	France	Bone tissue	IA_1	1-1-1-3-1-1-8-6	4	4	D1
HL082PA1	USA	Acne	IA_1	1-1-1-3-1-1-7-6	13	4	E5
HL110PA2	USA	Acne	IA_1	1-1-1-1-1-1-8-6	21	4	E3
409-HCl[c]	USA	ND	IA_1	1-1-1-3-1-1-8-41	133	4	D1
CC003-HC2[c]	USA	ND	IA_1	1-1-1-3-1-1-8-41	133	4	D1
SK187	USA	Skin	IA_1	16-1-1-15-1-4-8-6	19	S	E2
HL037PA1	USA	Skin	IA_2	1-1-1-5-1-4-8-2	2	2	F4
HL025PA2	USA	Skin	IA_2	1-1-1-5-1-4-8-2	2	2	F4
HL059PA2	USA	Skin	IA_2	1-1-1-5-1-4-8-2	2	2	F1
HL059PA1	USA	Skin	IA_2	1-1-1-5-1-4-8-2	2	2	F1
HL005PA4	USA	Skin	IA_2	1-1-1-5-1-4-8-2	2	2	F1
HL002PA1	USA	Acne	IA_2	1-1-1-5-1-4-8-2	2	2	F1
HL027PA1	USA	Skin	IA_2	1-1-1-5-1-4-8-2	2	2	F1
HL083PA2	USA	Acne	IA_2	1-1-1-5-1-4-8-2	2	2	F3
HL046PA1	USA	Acne	IA_2	1-1-1-5-1-4-8-2	2	2	F1
HL013PA1	USA	Acne	IA_2	1-1-1-5-1-4-8-2	2	2	F1
HL050PA3	USA	Skin	IA_2	1-1-1-5-1-4-8-2	2	2	F1
HL087PA1	USA	Acne	IA_2	1-1-1-5-1-4-8-2	2	2	F1
HL087PA3	USA	Acne	IA_2	1-1-1-5-1-4-8-2	2	2	F1
P.acn33	France	Endophthalmitis	IA_2	1-1-1-5-1-4-8-2	2	2	F1
HL067PA1	USA	Acne	IA_2	1-1-1-5-1-4-8-9	24	2	F2

P.acn31	France	Endophthalmitis	IA_2	1-1-1-13-1-4-8-2	36	2	F4
HL030PA2	USA	Skin	IA_2	1-1-1-5-3-5-8-7	22	S	F4
20.2.L1	Denmark	[10]	IA_2	1-1-1-5-3-5-8-7	22	S	F4
P.acn17	France	Keratitis	IA_2	1-1-1-5-3-5-8-7	22	S	F5
HL063PA2	USA	Skin	IA_2	1-18-1-5-3-5-8-8	23	S	F4
25.1.R1	Denmark	[10]	A_2	1-1-1-18-1-4-8-2	57	2	F1
HL050PA1	USA	Skin	IA_2	1-1-1-5-1-4-9-2	91	2	F1
HL030PA1	USA	Acne	IB	1-1-1-4-1-4-8-6	5	5	H1
KPA171202[d]	Germany	Contaminant	IB	1-1-1-4-1-4-8-6	5	5	H2
6609	Hungary	Skin	IB	1-1-1-4-1-4-8-6	5	5	H1
CCUG32901[e]	Sweden	Blood	IB	1-1-1-4-1-4-8-6	5	5	H1
434-HC2[c]	USA	ND	IB	1-1-1-4-1-4-8-6	5	5	H1
KPL2008[c]	USA	ND	IB	1-1-1-4-1-4-8-6	5	5	H1
LYR_BL	France	Bone tissue	IB	1-1-1-4-1-4-8-6	5	5	H1
NLAE-zl-G260	USA	ND	IB	1-1-1-4-1-4-8-6	5	5	H1
21.1.L1	Denmark	[10]	IB	1-1-12-4-1-4-8-6	56	5	H1
P6	Germany	Prostate	IB	1-1-1-4-1-4-29-6	118	5	H1
PA2	USA	Prostate	IB	1-1-13-4-1-4-8-6	119	5	H1
HL097PA1	USA	Acne	IC	9-1-4-8-6-8-14-14	70	107	G1
PRP-38	UK	Acne	IC	9-1-4-8-6-8-14-14	70	107	G1
PV66	UK	Acne	IC	9-1-5-8-6-8-14-6	85	107	G1
5-1-3	France	Acne	IC	9-1-4-8-6-8-14-6	107	107	G1
HL082PA2	USA	Acne	II	17-4-2-4-2-3-10-10	6	6	K6
HL060PA1	USA	Acne	II	17-4-2-4-2-3-10-10	6	6	K1
HL042PA3	USA	Acne	II	17-4-2-4-2-3-10-10	7	6	K2
HL110PA3	USA	Acne	II	15-4-2-4-2-3-10-10	7	6	K2
HL110PA4	USA	Acne	II	15-4-2-4-2-3-10-10	7	6	K2
HL103PA1	USA	Acne	II	17-9-2-4-2-3-10-10	25	6	K1
HL050PA2	USA	Skin	II	17-4-2-17-2-3-11-11	26	S	K4
J139	USA	Skin	II	17-4-2-16-2-12-10-12	28	72	K8
HL001PA1	USA	Skin	II	17-4-2-4-2-6-10-12	30	72	K1

ATCC11828[f]	USA	Abscess	II	17-4-2-4-9-12-10-13	27	S	K9
KCOM1861	Korea	Osteomyelitis	II	17-4-2-4-2-12-10-28	79	72	K2
KPL1847[c]	USA	ND	II	17-4-2-4-2-3-11-11	121	6	K4
KPL1849[c]	USA	ND	II	17-4-2-4-2-3-11-11	121	6	K4
PMH5	Denmark	PMH	III	7-6-3-7-5-9-13-16	33	77	L1
PMH7	Denmark	PMH	III	7-6-3-7-5-9-13-16	33	77	L1
Asn12	UK	Spinal disc	III	7-6-3-7-5-9-13-16	33	77	L1
Asn10	UK	Prosthetic hip joint	III	7-6-7-7-5-9-12-16	81	77	L4
					N=48		N=32

[a]Isolates comprising the HMP and other WGS projects listed at http://www.ncbi.nlm.nih.gov/genome/genomes/1140

[b]Type strain purchased from the National Collection of Type Cultures (Colindale, UK)

[c]HMP isolates classified as novel *Propionibacterium* spp, but clearly *P. acnes* based on MLST analysis

[d]Strain purchased from the German Collection of Microorganisms and Cell Cultures (DSMZ; Braunschweig, Germany)

[e]Strain from Culture Collection, University of Göthenburg

[f]Strain purchased from American Type Culture Collection

[g]Allelic profiles and STs assigned using database at http://pubmlst.org/pacnes/

[h]SLST types assigned using database at http://medbac.dk/slst/pacnes
